# Sponge-like nanoporous single crystals of gold

**DOI:** 10.1038/ncomms9841

**Published:** 2015-11-10

**Authors:** Maria Koifman Khristosov, Leonid Bloch, Manfred Burghammer, Yaron Kauffmann, Alex Katsman, Boaz Pokroy

**Affiliations:** 1 Department of Materials Science and Engineering, Technion—Israel Institute of Technology, Haifa 32000, Israel; 2 Russell Berrie Nanotechnology Institute, Technion—Israel Institute of Technology, Haifa 32000, Israel; 3 European Synchrotron Radiation Facility, BP 220, Grenoble F-38043, France; 4 X-ray Microspectroscopy and Imaging Research Group, Department of Analytical Chemistry, Ghent University, Ghent B-9000, Belgium

## Abstract

Single crystals in nature often demonstrate fascinating intricate porous morphologies rather than classical faceted surfaces. We attempt to grow such crystals, drawing inspiration from biogenic porous single crystals. Here we show that nanoporous single crystals of gold can be grown with no need for any elaborate fabrication steps. These crystals are found to grow following solidification of a eutectic composition melt that forms as a result of the dewetting of nanometric thin films. We also present a kinetic model that shows how this nano-porous single-crystalline structure can be obtained, and which allows the potential size of the porous single crystal to be predicted. Retaining their single-crystalline nature is due to the fact that the full crystallization process is faster than the average period between two subsequent nucleation events. Our findings clearly demonstrate that it is possible to form single-crystalline nano porous metal crystals in a controlled manner.

## Abstract (web summary)

Naturally occurring single crystals can exhibit various intricate porous morphologies. Here, the authors are able to grow nanoporous single crystals of gold following solidification of a eutectic composition melt that forms as a result of the dewetting of nanometric thin films.

## Graphical abstract

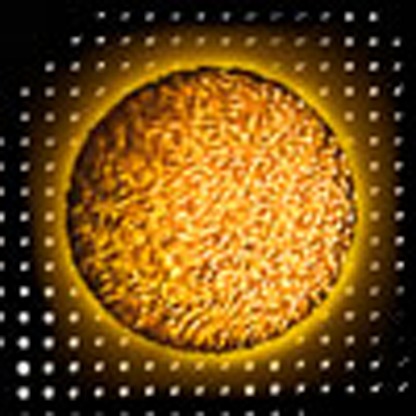

Crystals grown in the laboratory by classical methods of nucleation and growth have facets dictated by their atomic structure and by minimization of the surface free energy of the different crystallographic planes^1^. These features result in faceted crystals in which the revealed planes are the low-energy ones. Remarkably, however, biogenic crystals produced by living organisms often demonstrate unfaceted single crystals with rounded, intricate and even porous structures. Such single crystals can be produced via an amorphous precursor phase in which the precursors can be moulded into any desired shape before their crystallization^2^,^3^,^4^.

The achievement of intricately shaped nanoporous single-crystalline materials is undoubtedly of considerable scientific and technological potential^5^. It was previously shown that micrometre-sized porous single crystals of calcite can be grown via bio-inspired routes^6^,^7^. Even porous metal materials (PMMs) that are polycrystalline are functionally promising^8^,^9^ for research and industry in the fields of chemistry^10^, medicine^11^, environmental sciences, energy storage and others^12^. Besides the intrinsic properties of monolithic metals, in PMMs the new and attractive properties derived from the shape, size and distribution of pores include changes in specific surface area, permeability and capillarity to mention just a few. Owing to their unique structure, such porous metals are widely employed in catalytic support^13^, filtration^14^, separation^15^, heat exchange^16^, fuel cells^17^ and many additional uses. Common methods of preparing nanoporous metals are dealloying^18^, or depositing the metal within a three-dimensional (3D) mould^19^ such as a latex sphere^20^ or other soft templates^21^.

A particularly important example of a PMM is nanoporous gold, which has great potential for applications in catalysis^22^,^23^, sensors^24^, actuators^25^ and electrodes for electrochemical uses^26^. The yield strength of nanoporous gold is very high, equal to or higher than that of bulk Au, whereas the nanoporous material also has a much lower density and very large surface area^27^. A common way to obtain nanoporous gold is by dealloying of an Au-Ag alloy via selective removal of Ag in a corrosive environment such as a nitric acid solution^28^.

The nanoporous gold prepared by dealloying has a nanocrystalline structure^29^. Formation of nanoporous gold from an original Au-Ag micrometre-sized grained ingot has been reported^30^, but it was prepared by dealloying. This result can be clearly observed from the decrease in X-ray coherence length, observed in the broadening of the diffraction peaks and the increase in lattice misorientations. A few examples of single-crystalline porous gold have been described, but these nanometric structures were only two-dimensional^31^. Besides possessing the attractive properties of polycrystalline porous gold, a nanoporous single crystal of gold is likely to have additional novel properties. Studies have shown that grain boundaries in thin films of gold enhance the electrical resistivity of nanometric thick films owing to grain boundary-induced scattering of the electrons^32^,^33^,^34^. Not only would the conductivity of the porous single crystal be higher than that of porous polycrystalline gold, but as single crystals their 3D crystallographic orientation could also be selected. The higher conductivity expected from such nanoporous structures could increase the efficiency of electrodes for electrochemical supercapacitors, as described elsewhere^26^. Another advantage of the absence of grain boundaries in such structures is to be found in the higher thermal stability of their micro- and nano-framework. This results from lower rates of self-diffusion, which are attributable in turn to the total elimination of grain boundary diffusion routes. The latter adventitious property could be helpful in cases where such structures are utilized in catalysis when operational temperatures in the range of 20−200 °C are needed^35^,^36^. As one of the most prevalent applications of nanoporous gold is in catalysis, we feel that this property could be of interest to others working in this field.

In this paper, we present a simple method to grow micrometre-sized nanoporous single crystals of gold with intricate morphologies. Unlike any other examples to date, our structures are formed via thermodynamically driven self-formation. Thin films of two components are evaporated onto a nonreactive surface, followed by heating to above the eutectic temperature. This procedure causes the films to melt, producing a eutectic melt on the surface. Because of the low surface energy of the appropriately chosen substrate, dewetting takes place and isolated droplets of the melt are produced all over the substrate's surface. Fast cooling preserves the shapes of both the droplets and the eutectic microstructure. Finally, by selectively etching one of the components, a porous single-crystal structure of the other component is obtained. We demonstrate this general principle on an Au-Ge eutectic system, which yields nanoporous single crystals of gold. Furthermore, we present a model that explains our experimental findings and enables us to calculate the conditions needed to obtain rationally designed single-crystalline porous structures. It also enables us to predict the size limits of such an approach.

## Results

### Preparation process description

**Figure 1:**
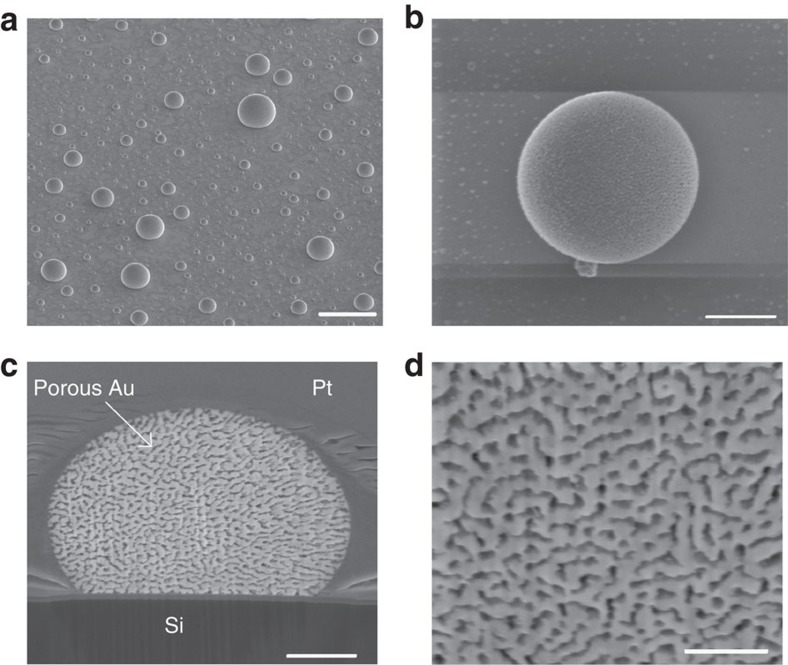
Nanoporous single-crystal particles of gold. (**a**) High-resolution scanning electron microscopy (HRSEM) micrograph of the droplets on a SiO_2_ surface, large area view (scale bar, 20 μm). (**b**) HRSEM micrograph of a eutectic gold droplet after wet etching, top view (scale bar, 2 μm). (**c**) HRSEM micrograph of a cross-section of the droplet shown in **b**, obtained using a focused ion beam (FIB), reveals the nano-porous structure of gold after wet etching of Ge (scale bar, 1 μm). The droplet was covered with Pt in order to protect it during the ion beam etching. (**d**) HRSEM micrograph of high magnification in **c** (scale bar, 500 nm).

Thin films comprising two layers were evaporated at relative thicknesses appropriate for the eutectic ratio, which in the Au-Ge system is 28 at% Ge (ref. 37). Annealing above the eutectic temperature resulted in melting of these thin films and formation of a eutectic melt. Choice of SiO_2_ as a substrate enabled droplets of this melt to be formed by dewetting. Cooling to below the eutectic temperature resulted in solidification of the eutectic liquid to form a eutectic-like solid structure. Wet etching of the samples to remove the germanium revealed droplets of porous gold, as seen in Fig. 1a,b by high-resolution scanning electron microscopy (HRSEM). Analysis of the microstructure by energy-dispersive X-ray spectroscopy confirmed that the Ge was fully removed during the etching process (Supplementary Table 1). Using a focused ion beam (FIB), we were able to visualize a cross-section of the droplets and obtain a view of the gold microstructure within a droplet (Fig. 1c,d).

### TEM diffraction of the gold nanoporous single crystal

**Figure 2:**
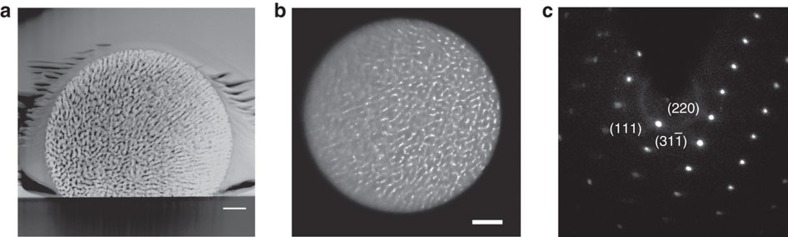
TEM investigation of a nanoporous single crystal. (**a**) A high-angle annular dark-field scanning TEM (HAADF-STEM) micrograph of a cross-section of a droplet, showing microstructure of gold after Ge etching (scale bar, 500 nm). (**b**) TEM image of the area of the droplet from which the diffraction pattern was acquired, showing the selected area aperture in a (scale bar, 500 nm). (**c**) Diffraction pattern taken from the area shown in **b**, fully indexed within the gold structure; zone axis [121].

A thin cross-section of a sample droplet was prepared by FIB and examined by transmission electron microscopy (TEM). A high-angle annular dark-field scanning TEM micrograph of the cross-section is presented in Fig. 2a. Selected area diffraction on an area with a diameter of approximately 4 μm in the centre (the main part) of the droplet (Fig. 2b) revealed a diffraction pattern characteristic of a single crystal that could be fully indexed within the gold structure (Fig. 2c). This indicates that the gold in the eutectic-like structure within the droplet had solidified into an intricately shaped single crystal.

### Synchrotron scanning diffractometry

**Figure 3:**
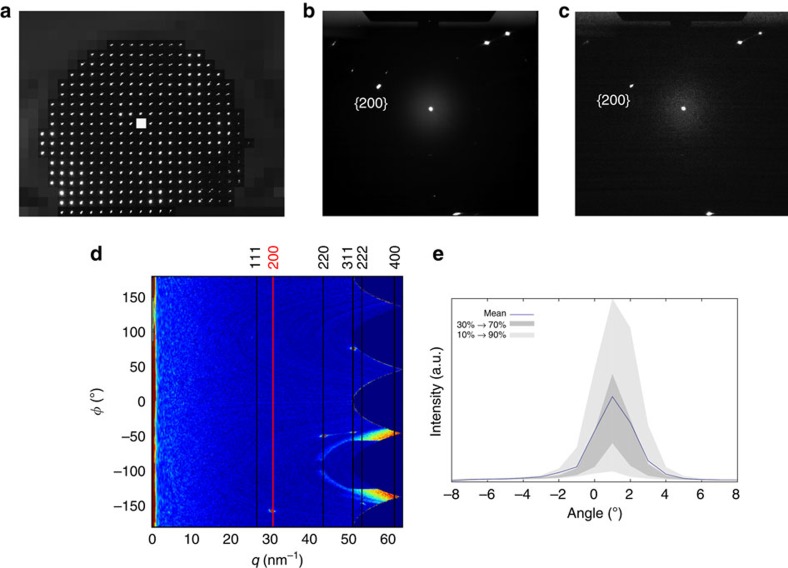
Synchrotron submicron scanning diffractometry of the gold nanoporous single crystal. (**a**) Reflection map for the {200} planes of the sample shown in Fig. 2. (**b**) The average diffraction pattern from the drop area. (**c**) Diffraction pattern from the centre of the drop, marked with a white square on **a**. (**d**) Azimuthally regrouped central diffraction pattern, *q*=2π/*d*, *ϕ*—radial axis. (**e**) Rocking curve of a reflection from the {200} planes.

To further verify that this porous gold is indeed a single crystal, we utilized another state-of-the-art characterization technique, namely synchrotron submicron scanning diffractometry (ID13; European Synchrotron Research Facility (ESRF), Grenoble, France), on a FIB-sectioned gold crystal. The same cross-section as that of the droplet investigated by TEM (Fig. 2) was now examined by scanning diffraction. Diffraction patterns were scanned over the entire cross-section of the droplet (Fig. 3). Figure 3a depicts a map of a single reflection ({200} plane, 0° rotation). The same reflection occurs throughout the droplet at nearly the same radial and azimuthal coordinates, with only some slight variations in intensity. Figure 3b depicts the average of all the diffraction scans taken from the entire area of the droplet. This can be compared with Fig. 3c, which shows a single diffraction from the droplet's centre. From a comparison of these images, it is evident that the major reflections are identical, and do not shift their positions even though the figure integrates a large number of individual scans (317 in all). Figure 3d represents the azimuthally regrouped central diffraction with markings for gold crystallographic planes, and Fig. 3e depicts a rocking curve for the same reflection. Shown are the mean intensity over the droplet and its 10, 30, 70 and 90% percentiles. Results for the {220} planes are shown in Supplementary Fig. 1. The results obtained from the TEM and the scanning diffractometry provide conclusive proof that the nanoporous gold crystals are indeed single crystals.

### Controlling the micro- and nanostructure

**Figure 4:**
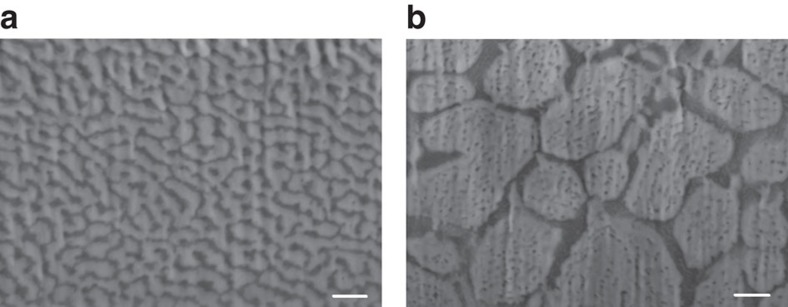
Controlling the micro- and nanostructures of the single-crystal nanoporous gold. HRSEM micrograph of a FIB cross-section of the eutectic microstructure at (**a**) 35 °C s^−1^ and (**b**) 0.6 °C s^−1^. Scale bar, 200 nm.

By altering the cooling rates of the samples, micro- and nanostructures of different sizes could be obtained (Fig. 4). Ligament sizes in the solid droplet after cooling at a fast rate (35 °C s^−1^) were about 57±12 nm for gold and 43±8 nm for germanium (Fig. 4a). At a slow cooling rate (0.6 °C s^−1^), the approximate ligament sizes of the gold and the germanium were 300 nm (or larger) and 39±8 nm, respectively (Fig. 4b). This phenomenon resulted from a coarsening of the solid eutectic phase during the longer cooling phase.

### Surface area and thermal stability

In addition, by analysing the cross-sectional HRSEM micrographs, we estimated the surface area of the porous Au crystals. For this purpose, we assumed a homogeneous microstructure and used a previously described methodology^38^. Our calculations yielded an estimated surface area of 3.1 m^2^ g^−1^, which is in the range of other known nanoporous metals^39^,^40^,^41^ approximately 3–10 m^2^ g^−1^.

We then compared the thermal stability of our single-crystalline nanoporous nanostructures to that of nanoporous gold prepared by the dealloying method^28^. The results are shown in Supplementary Figs 2–5. After annealing both types of porous gold samples, one prepared by our eutectic decomposition method as described here, and the other by dealloying, at 200 °C for 15 min and for 45 min, and at 250 °C for 15 min, we compared their nanostructures. The samples prepared by the dealloying method failed at lower treatment time and temperatures: grain boundaries developed into cracks, porous particles were found to be damaged at the edges and coarsening was clearly observed. In conspicuous contrast, samples prepared by our method retained their porous nanostructure and showed no evidence of damage.

## Discussion

By using state-of-the-art characterization techniques such as TEM and synchrotron submicrometre scanning diffractometry, we were able to show that the porous gold structure within the eutectic droplets is a single crystal. Use of these techniques further revealed continuous growth of a single gold crystal during formation of the eutectic-like two-phase structure during its solidification. Although nanoparticles of nanoporous gold have been demonstrated previously^19^,^20^,^21^,^28^, our method is novel in that we do not use the dealloying mechanism (a ‘top-down' approach); instead, our porous single crystals grow from a liquid phase during eutectic decomposition, which is a spontaneous ‘bottom-up' approach. Growth of the porous single crystal by the latter method occurs rapidly and is a self-forming process (eutectic decomposition). The outcome is a nanoporous single crystal of gold, with the germanium residing only in the pores. Another advantage of our method is that by means of a simple, fast and low-cost process we obtain free-standing porous particles whose shape can be determined by the original droplets wetting properties and which can easily be used in other processes with no need to cut the bulk material by any type of fabrication technique.

When comparing our method with the conventional dealloying procedure in the formation of nanoporous gold, it should be borne in mind that not all that appears to be a single crystal is indeed a single crystal. Several studies^30^,^42^ have implied that given the apparently unchanged morphology and orientation of the original grains before and after dealloying, the nanoporous gold products are still single crystals of the same original size. This was demonstrated on the basis of grain morphology and electron back-scattered diffraction in SEM. However, the only way to unambiguously prove that a grain is in fact a single crystal is by the use of high-resolution X-ray diffraction techniques, as we did in this study. For further verification, we performed *in-situ* dealloying with high-resolution powder diffraction at the synchrotron (ID22; ESRF) on AuAl_2_ intermetallic. The results clearly showed how the coherence length of the gold that remains after dealloying is drastically reduced (Supplementary Fig. 6). Although the structure of AuAl_2_ is different than that of pure gold (both are cubic), the literature shows the exact same phenomenon when dealloying silver from gold^30^.

One of the nano porous' gold promising applications is a low-temperature catalytic CO oxidation^35^,^36^ to CO_2_. This process can be undertaken at temperatures in the range of 20–200 °C, and usually is performed utilizing nanoporous gold prepared by dealloying or gold nanoparticles. The disadvantage of the nanoporous gold prepared by dealloying is coarsening of the nanoporous structure because of the temperature-induced diffusion. Although our samples have slightly larger ligament size as compared with the ligament size of the dealloyed gold counterpart, and therefore slightly lower surface area, the former demonstrate superior thermal stability. This relative improvement is due to the lack of grain boundaries within single crystals.

Discerning the mechanism governing the growth of nanoporous crystals that maintain the characteristics of a single crystal is important not only for basic scientific knowledge but also to allow this technique to be applied, in a controlled manner, in technologically important crystalline systems. We therefore felt it necessary to develop a kinetic model that could explain how these nanoporous single crystals are formed, and would allow us to estimate the maximal achievable size of such crystals.

In the case of a fully eutectic structure, the volume ratio *V*_Au_/*V*_Ge_=1.92 (calculated on the basis of the eutectic composition) enables gold crystals to grow continuously throughout the entire droplet, accompanied by repeated nucleation and growth of rod-like germanium crystal channels surrounded by a gold matrix. When considering a crystallization process in the Au-Ge eutectic system, it should be remembered that normal crystallization of a non-equilibrium eutectic liquid usually starts with the heterogeneous nucleation of one solid phase, in the present case probably Au. The nucleation also most probably takes place at the liquid/substrate interface. In the next stage, the adjacent liquid is substantially enriched by Ge, providing heterogeneous and, if possible, epitaxial nucleation of Ge crystals at the surface of this Au crystal. The formation is governed by the free energy gain as a result of undercooling to below the eutectic temperature, Δ*T*=(*T*_eut_–*T*), while atoms are being transferred from the liquid phase to the growing crystal:


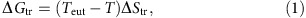


where Δ*S*_tr_ is the entropy change in the *L* (eutectic) → Au(s) + Ge(s) transformation. Obviously, additional energy is needed to create the solid/liquid and Au(s)/Ge(s) interfaces. The epitaxial nucleation and growth of Ge on Au are possible in the crystallographic orientation relationships 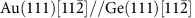 and 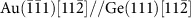
43, which may provide relatively low interfacial energies. Growth of the eutectic structure is limited by volume diffusion in the liquid in front of the freezing solid/liquid interface. The growth rate can be evaluated according to the simple model suggested by Turnbull^44^:


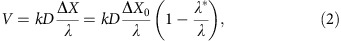


where *D* is the diffusion coefficient in the liquid, Δ*X* is the difference in composition of the liquid above the freezing interface across the eutectic lamellar spacing λ, Δ*X*_*0*_ is the difference in composition for λ→∞, *k*∼1 is a geometry-dependent coefficient and λ* is the critical lamellar spacing defined by Zener^45^:


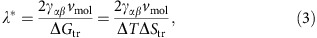


where *γ*_αβ_ is the energy of the solid/solid (Au/Ge) interface and *v*_mol_ is the liquid molar volume. Using the transformation entropy value for the Au-Ge system^46^ Δ*S*_tr_= 23.9 J mol^−1^ K^−1^, and assuming *γ*_αβ_=(0.2÷0.4) J m^−2^ and Δ*T*=(10÷20) K, we can estimate from equation (3) the critical width of lamellae as λ*=(8÷32) nm, which is comparable to the half of the observed ligament size (40÷60) nm. The relatively minor observed supercooling is attributable to the heterogeneous character of the nucleation. The supercooling corresponds to a difference in composition of the couple zone of the Au/Ge phase diagram^46^ Δ*X*_0_=0.01÷0.02. From viscosity experiments at high temperatures^47^, the self-diffusion coefficient in the liquid gold was found to be in the range of 2.02 × 10^−9^ to 3.35 × 10^−9^ m^2^ s^−1^ at temperatures between 1,063 and 1,364 °C with an activation energy of *E*_a_=0.316 eV. The diffusion coefficient in the undercooled eutectic liquid can be evaluated by extrapolation of these values to temperatures below the Au\Ge eutectic temperature. *D*=*D*_0_ exp (−*E*_a_/*k*_B_*T*) with *D*_0_=3.1 × 10^−8^ m^2^ s^−1^, yielding ∼8.5 × 10^−11^ m^2^ s^−1^ for the eutectic temperature. We can now estimate the growth rate and the time to achieve full crystallization of the eutectic structure. The growth rate is evaluated as *V*=(10÷30) μm s^−1^ and the total crystallization time in droplets (2÷5) μm in size is *τ*_c_=*R*_d_*/V*=(0.07÷0.5) s, where *R*_d_ is the droplet size.

A more sophisticated theory of steady-state eutectic growth was developed by Jackson and Hunt^48^ and then modified by several authors^49^,^50^,^51^. The estimations based on this theory are presented in Supplementary Note 1. According to evaluations based on the Jackson and Hunt theory, the freezing rates, *V*=(30÷60) μm s^−1^, are slightly higher (and the total crystallization times are lower) than those obtained from the Turnbull model^44^.

The rate of heterogeneous nucleation of gold crystals, presumably at the substrate/liquid interface, can be deduced on the basis of classical kinetic theory^52^,^53^:


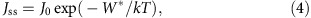


where W* is the height of the heterogeneous nucleation barrier and *J*_0_ is a pre-exponential factor. As shown in Supplementary Note 2 and Supplementary Fig. 8, for reasonable material parameters, the pre-exponential factor can be evaluated as *J*_0_ ≈ (4÷6) × 10^20^ s^−1^ μm^−3^, and at the moment when the first stable nucleus appears, the heterogeneous nucleation rate in the droplet reaches 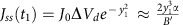 nuclei per second (see Supplementary Note 2 for details), which for the cooling rate *α*=1 C° s^−1^ (used in most of the present experiments) provides about (0.8÷1.0) nuclei per second. Comparison of the average time 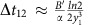 between the two first nucleation events with the time of full crystallization of the droplet *τ*_c_ allows us to formulate a criterion for the appearance of a monocrystal porous structure described in the previous sections:


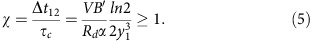


For typical values of parameters (see Supplementary Tables 2 and 3, and Supplementary Note 2 for details): *B*′=700 °C, *y*_1_=6.5, 
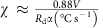
. For droplets with radii *R*_d_≤10 μm and cooling rates ≤1 °C s^−1^, with *V*=(30÷50) μm s^−1^, formation of Au-Ge eutectics with the monocrystal gold matrix seems to be highly probable. On the other hand, with faster cooling, for example with the rate *α*=35 °C s^−1^, such a structure is unlikely to be formed. In other words, for cooling rates ∼1 °C s^−1^ the second nucleus appears (0.7÷0.9) s on average after the first one, and this period is long enough to allow full crystallization of the (2÷10) μm droplets before the second nucleation event occurs. At the same time, for droplets with radii *R*≥20 μm and/or high cooling rates *α*≥10 °C s^−1^, several nuclei can appear during crystallization (for details, see Supplementary Fig. 7).

In conclusion, in each droplet during cooling of the sample, two processes are competing—nucleation and crystal growth. The times taken for the full crystallization process in the micrometre-sized droplets investigated here appeared to be substantially shorter than the average period between the two subsequent events of nucleation of gold crystals from the eutectic melt. This can explain why the gold matrix of the eutectic structure was formed as a single crystal. To further validate our kinetic model, we studied how changing the cooling rates of the process affects the number of single-crystal domains in each droplet. As our model suggests, an increase in the cooling rate from 0.6 to 35 °C s^−1^ is followed by a clear transition from single crystal to polycrystalline droplets (Supplementary Fig. 7).

To conclude, using the relatively simple method of dewetting from thin films, and by exploiting thermodynamic phenomena such as crystallization from a eutectic melt, we demonstrated here that it is possible to produce nanoporous gold single crystals. We have developed a kinetic model that explains this phenomenon and shows that the full crystallization process is faster than the average period between two subsequent nucleation events, a key factor allowing the intricate 3D single crystals of gold to retain their single-crystalline nature. Based on our calculations, we can predict that this method allows for the growth of nanoporous single crystals of gold of up to several hundred micrometres in size. We also clearly showed that nanoporous single crystal prepared by eutectic composition demonstrate superior thermal stability as compared with their counterpart nanoporous gold prepared by dealloying, which is essential for catalysis. We believe that this method can also be employed in other crystal systems, thereby opening the door to new technological capabilities.

## Methods

### Sample preparation

SiO_2_ (100 nm) was grown on (001) Si wafers by thermal oxidation at 1,100 °C. The oxide layer served as a diffusion barrier for preventing Si migration from the wafer. Gold and germanium films (99.999% pure, Sigma-Aldrich) were successively evaporated onto the SiO_2_ substrate in an e-beam-equipped AircoTemescal FC-1800 evaporating system under a high vacuum of 10^−7^ Torr at room temperature, yielding a deposition rate of 8 Å s^−1^. The gold and germanium films were 150 and 78 nm thick, respectively. Samples were thermally annealed at 550 °C for 5 min in a MILA-5000 ULVAC-RIKO rapid thermal annealer or in a Jiplec JetFirst-100 rapid thermal annealer in an ambient flow of Ar-H_2_ (15% H_2_; 99.999%, 150 s.c.c.m.) or vacuum (10^−5^ Torr) at a heating rate of 10 °C s^−1^. The cooling rates were 35 and 0.6 °C s^−1^. Samples were wet-etched in two steps: (i) immersion in a solution of NH_4_OH:H_2_O_2_ (1:25% vol) for 1 h and then rinsed, first in deionized water and then in ethanol; (ii) immersion in KOH solution (1.25 M) for 16 h and then rinsed again in water and ethanol. Cross-sectional samples were prepared by an FEI Strata 400S dual-beam FIB. Low-voltage argon ion milling was then applied for final thinning and cleaning of surface using the Gentle Mill, model IV8 (Technoorg Linda). Thermal stability experiments were performed in Jiplec JetFirst-100 rapid thermal annealer in 200–250 °C for altering times in vacuum (10^−5^ Torr).

### Sample characterization

Surfaces were imaged with a Zeiss Ultra Plus HRSEM, combined with energy-dispersive X-ray spectroscopy. Images of the cross-sections were acquired with the Strata 400S dual-beam FIB. TEM imaging and electron diffractions were obtained using an FEI C_s_ corrected Titan 80–300 KeV FEG-S/TEM operated at 300 KeV. Single-crystalline droplets were characterized by nanofocus X-ray beam analysis on ID13 of the ESRF . The samples were rotated from −45° to 45°, with 1° intervals. After each interval, the sample was scanned with an X-ray beam at a wavelength of 0.83189 Å, focused to approximately 200 × 150 nm (full-width at half-maximum) at the sample plane.

## Additional information

**How to cite this article:** Khristosov, M. K. *et al.* Sponge-like nanoporous single crystals of gold. *Nat. Commun.* 6:8841 doi: 10.1038/ncomms9841 (2015).

## Supplementary Material

Supplementary Information Supplementary Figures 1-8, Supplementary Tables 1-3, Supplementary Notes 1-2 and Supplementary References

## References

[b1] Vlachos D. G., Schmidt L. D. & Aris R. Kinetics of faceting of crystals in growth, etching, and equilibrium. Phys. Rev. B 47, 4896 (1993). 10.1103/physrevb.47.4896 10006649

[b2] Weiner S., Sagi I. & Addadi L. Choosing the crystallization path less traveled. Science 309, 1027–1028 (2005). 16099970 10.1126/science.1114920

[b3] Donnay G. & Pawson D. L. X-ray diffraction studies of echinoderm plates. Science (New York, NY) 166, 1147 (1969). 10.1126/science.166.3909.1147 17775574

[b4] Raup D. M. Crystallography of echinoid calcite. J. Geol. 67, 661–674 (1959).

[b5] Kollek T. *et al.* Porous and shape-anisotropic single crystals of the semiconductor perovskite CH3NH3PbI3 from a single-source precursor. Angew. Chem. Int. Ed. 54, 1341–1346 (2015). 10.1002/anie.201408713 25470357

[b6] Aizenberg J., Muller D. A., Grazul J. L. & Hamann D. Direct fabrication of large micropatterned single crystals. Science 299, 1205–1208 (2003). 12595685 10.1126/science.1079204

[b7] Park R. J. & Meldrum F. C. Synthesis of single crystals of calcite with complex morphologies. Adv. Mater. 14, 1167–1169 (2002).

[b8] Ashby M. F. Metal Foams: a Design Guide Butterworth-Heinemann (2000).

[b9] Dumée L. F. *et al.* The fabrication and surface functionalization of porous metal frameworks–A review. J. Mater. Chem. A 1, 15185–15206 (2013).

[b10] Shakeri M. *et al.* Engineering and sizing nanoreactors to confine metal complexes for enhanced catalytic performance. ACS Catal. 4, 3791–3796 (2014).

[b11] Müller D. W., Matz A. M. & Jost N. Casting open porous Ti foam suitable for medical applications. Bioinspired, Biomim. Nanobiomaterials 2, 76–83 (2012).

[b12] Yuan W., Tang Y., Yang X. & Wan Z. Porous metal materials for polymer electrolyte membrane fuel cells–a review. Appl. Energy 94, 309–329 (2012).

[b13] Shigarov A. B., Kireenkov V. V., Kuzmin V. A., Kuzin N. A. & Kirillov V. A. Autothermal reforming of diesel fuel in a structured porous metal catalyst: Both kinetically and transport controlled reaction. Catal. Today 144, 341–349 (2009).

[b14] Liu W. & Canfield N. Development of thin porous metal sheet as micro-filtration membrane and inorganic membrane support. J. Membr. Sci. 409, 113–126 (2012).

[b15] Ryi S.-K. *et al.* Development of a new porous metal support of metallic dense membrane for hydrogen separation. J. Membr. Sci. 279, 439–445 (2006).

[b16] Calmidi V. & Mahajan R. Forced convection in high porosity metal foams. J. Heat Transf. 122, 557–565 (2000).

[b17] Moghaddam S. *et al.* An inorganic–organic proton exchange membrane for fuel cells with a controlled nanoscale pore structure. Nat. Nanotechnol. 5, 230–236 (2010). 20173756 10.1038/nnano.2010.13

[b18] Forty A. Corrosion micromorphology of noble metal alloys and depletion gilding. Nature 282, 597–598 (1979).

[b19] Martin C. R. Nanomaterials--a Membrane-Based Synthetic Approach DTIC Document (1994). 10.1126/science.266.5193.1961 17836514

[b20] Velev O., Tessier P., Lenhoff A. & Kaler E. Materials: a class of porous metallic nanostructures. Nature 401, 548–548 (1999).

[b21] Li C. *et al.* Electrochemical synthesis of mesoporous gold films toward mesospace-stimulated optical properties. Nat. Commun. 6, 6608 (2015). 25799072 10.1038/ncomms7608 PMC4382992

[b22] Ding Y., Kim Y. J. & Erlebacher J. Nanoporous gold leaf:‘Ancient technology'/advanced material. Adv. Mater. 16, 1897–1900 (2004).

[b23] Zielasek V. *et al.* Gold catalysts: nanoporous gold foams. Angew. Chem. Int. Ed. 45, 8241–8244 (2006). 10.1002/anie.200602484 17099919

[b24] Hieda M. *et al.* Ultrasensitive quartz crystal microbalance with porous gold electrodes. Appl. Phys. Lett. 84, 628–630 (2004).

[b25] Kramer D., Viswanath R. N. & Weissmüller J. Surface-stress induced macroscopic bending of nanoporous gold cantilevers. Nano Lett. 4, 793–796 (2004).

[b26] Lang X., Hirata A., Fujita T. & Chen M. Nanoporous metal/oxide hybrid electrodes for electrochemical supercapacitors. Nat. Nanotechnol. 6, 232–236 (2011). 21336267 10.1038/nnano.2011.13

[b27] Biener J. *et al.* Size effects on the mechanical behavior of nanoporous Au. Nano Lett. 6, 2379–2382 (2006). 17034115 10.1021/nl061978i

[b28] Wang D. & Schaaf P. Nanoporous gold nanoparticles. J. Mater. Chem. 22, 5344–5348 (2012).

[b29] Hodge A. *et al.* Monolithic nanocrystalline Au fabricated by the compaction of nanoscale foam. J. Mater. Res. 20, 554–557 (2005).

[b30] Petegem S. V. *et al.* On the microstructure of nanoporous gold: an x-ray diffraction study. Nano Lett. 9, 1158–1163 (2009). 19193021 10.1021/nl803799q

[b31] Tong W., Yang S. & Ding B. UV irradiation induced formation of single-crystal gold nanonetworks with controllable pore distribution. Colloids Surf. A Physicochem. Eng. Asp. 340, 131–134 (2009).

[b32] De Vries J. Resistivity of thin Au films as a function of grain diameter and temperature. J. Phys. F Metal Phys. 17, 1945 (1987).

[b33] Zhang Q., Cao B., Zhang X., Fujii M. & Takahashi K. Influence of grain boundary scattering on the electrical and thermal conductivities of polycrystalline gold nanofilms. Phys. Rev. B 74, 134109 (2006).

[b34] Wang H.-D., Liu J.-H., Zhang X., Guo Z.-Y. & Takahashi K. Experimental study on the influences of grain boundary scattering on the charge and heat transport in gold and platinum nanofilms. Heat Mass Transf. 47, 893–898 (2011).

[b35] Li D., Zhu Y., Wang H. & Ding Y. Nanoporous gold as an active low temperature catalyst toward CO oxidation in hydrogen-rich stream. Sci. Rep. 3, 3015 (2013). 24145317 10.1038/srep03015 PMC3804851

[b36] Wittstock A. *et al.* Nanoporous Au: an unsupported pure gold catalyst? J. Phys. Chem. C 113, 5593–5600 (2009).

[b37] Okamoto H. & Massalski T. B. Bull. Alloy. Phase Diagrams 5, 492–503 (1984).

[b38] Detsi E. *et al.* On the specific surface area of nanoporous materials. Acta Mater. 59, 7488–7497 (2011).

[b39] Tan Y. H. *et al.* Surface area and pore size characteristics of nanoporous gold subjected to thermal, mechanical, or surface modification studied using gas adsorption isotherms, cyclic voltammetry, thermogravimetric analysis, and scanning electron microscopy. J. Mater. Chem. 22, 6733–6745 (2012). 22822294 10.1039/C2JM16633J PMC3399672

[b40] Shulga O. V. *et al.* Preparation and characterization of porous gold and its application as a platform for immobilization of acetylcholine esterase. Chem. Mater. 19, 3902–3911 (2007). 18820734 10.1021/cm070238n PMC2553220

[b41] Ji C. & Searson P. C. Synthesis and characterization of nanoporous gold nanowires. J. Phys. Chem. B 107, 4494–4499 (2003).

[b42] Parida S. *et al.* Volume change during the formation of nanoporous gold by dealloying. Phys. Rev. Lett. 97, 035504 (2006). 16907511 10.1103/PhysRevLett.97.035504

[b43] Sayed S. Y. & Buriak J. M. Epitaxial growth of nanostructured gold films on germanium via galvanic displacement. ACS Appl. Mater. Interfaces 2, 3515–3524 (2010). 21105725 10.1021/am100698w

[b44] Turnbull D. Theory of cellular precipitation. Acta Metall. 3, 55–63 (1955).

[b45] Zener C. Kinetics of the decomposition of austenite. Trans. Aime 167, 550–595 (1946).

[b46] Wang J., Leinenbach C. & Roth M. Thermodynamic modeling of the Au–Ge–Sn ternary system. J. Alloys and Compd 481, 830–836 (2009).

[b47] Ofte D. The viscosities of liquid uranium, gold and lead. J. Nuclear Mater. 22, 28–32 (1967).

[b48] Jackson K. A. & Hunt J. D. The dendrite eutectic transition. Trans. Metall. Soc. AIME 23B, 1129 (1966).

[b49] Trivedi R., Magnin P. & Kurz W. Theory of eutectic growth under rapid solidification conditions. Acta Metall. 35, 971–980 (1987).

[b50] Kurz W. & Trivedi R. Eutectic growth under rapid solidification conditions. Metall. Trans. A 22, 3051–3057 (1991).

[b51] Karma A. Phase-field model of eutectic growth. Phys. Rev. E 49, 2245 (1994). 10.1103/physreve.49.2245 9961466

[b52] Kelton K. Crystal nucleation in liquids and glasses. Solid State Phys. 45, 75–177 (1991).

[b53] Debenedetti P. G. Metastable Liquids: Concepts and Principles Princeton Univ. (1996).

