# Supplementary material for: Sponge-like nanoporous single crystals of gold
Source: Nat Commun. 2015 Nov 10;6:8841. doi: 10.1038/ncomms9841 (PMC5426522; doi:10.1038/ncomms9841)
Supplement: Supplementary Information — Supplementary Figures 1-8, Supplementary Tables 1-3, Supplementary Notes 1-2 and Supplementary References [file ncomms9841-s1.pdf]

Supplementary Information

a

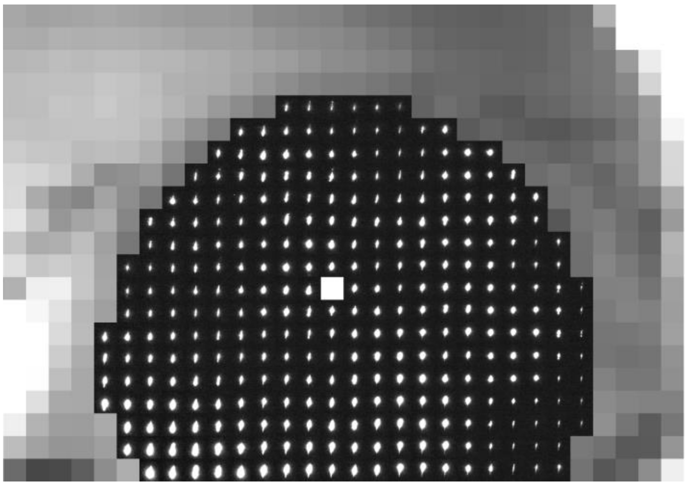

b

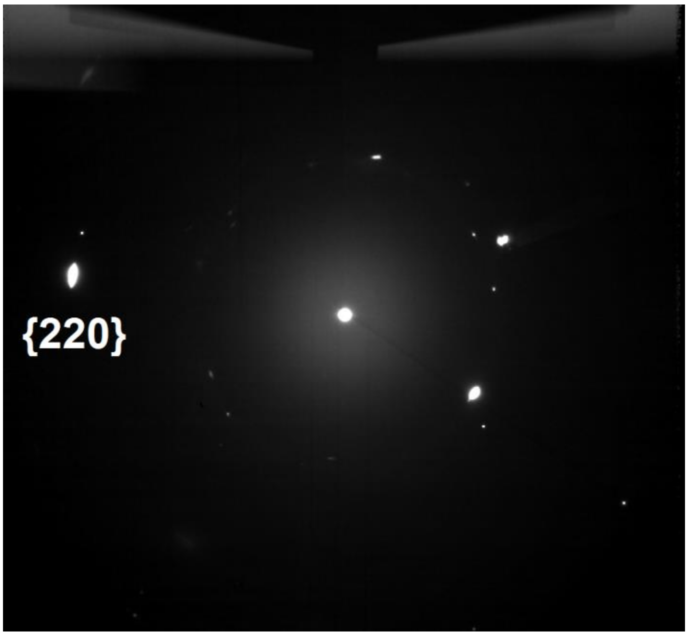

c

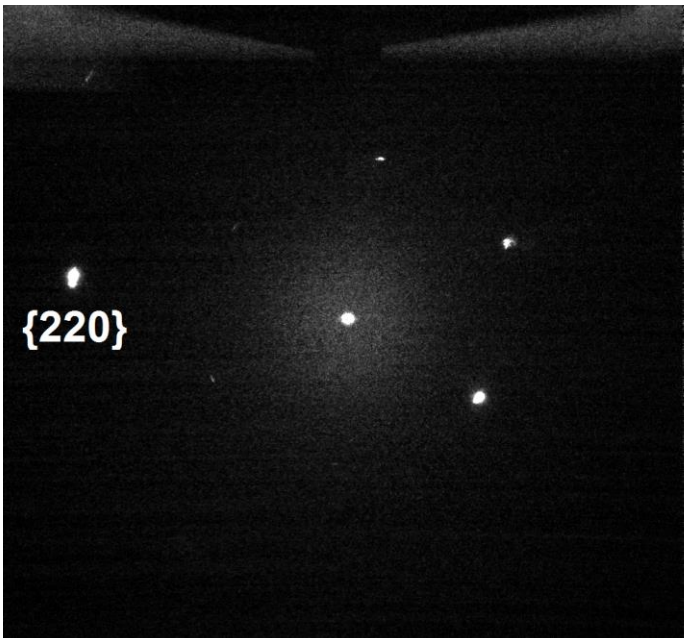

d **e**

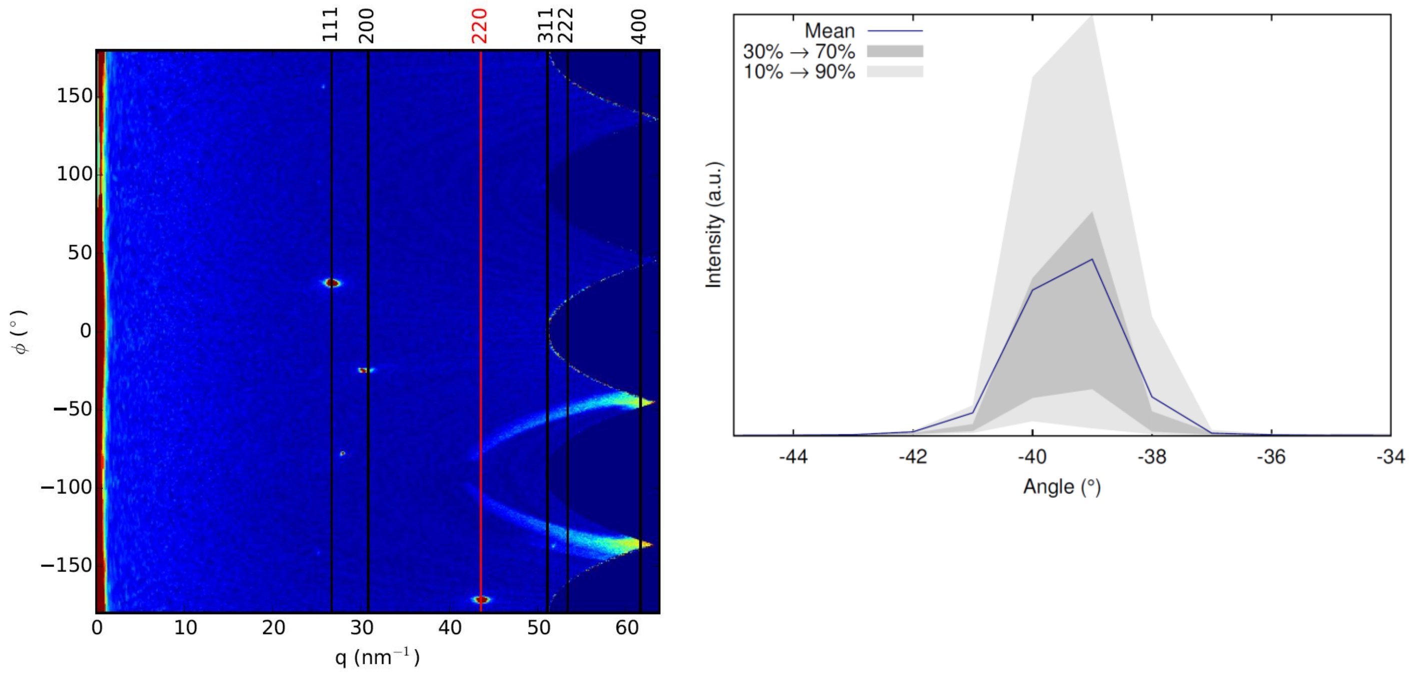

Supplementary Figure 1 Synchrotron submicron scanning diffractometry of the gold nanoporous single crystal. (**a)** Reflection map for the {220} planes (of the sample shown inFig. 2). (**b)** Average of diffraction patterns from the drop area. (**c)** Diffraction pattern from the center of the drop, marked with a white square on **a**. (**d)** Azimuthally regrouped central diffraction pattern, q=2π/d, φ – radial axis. (**e)** Rocking curve of reflections from the {220} planes.

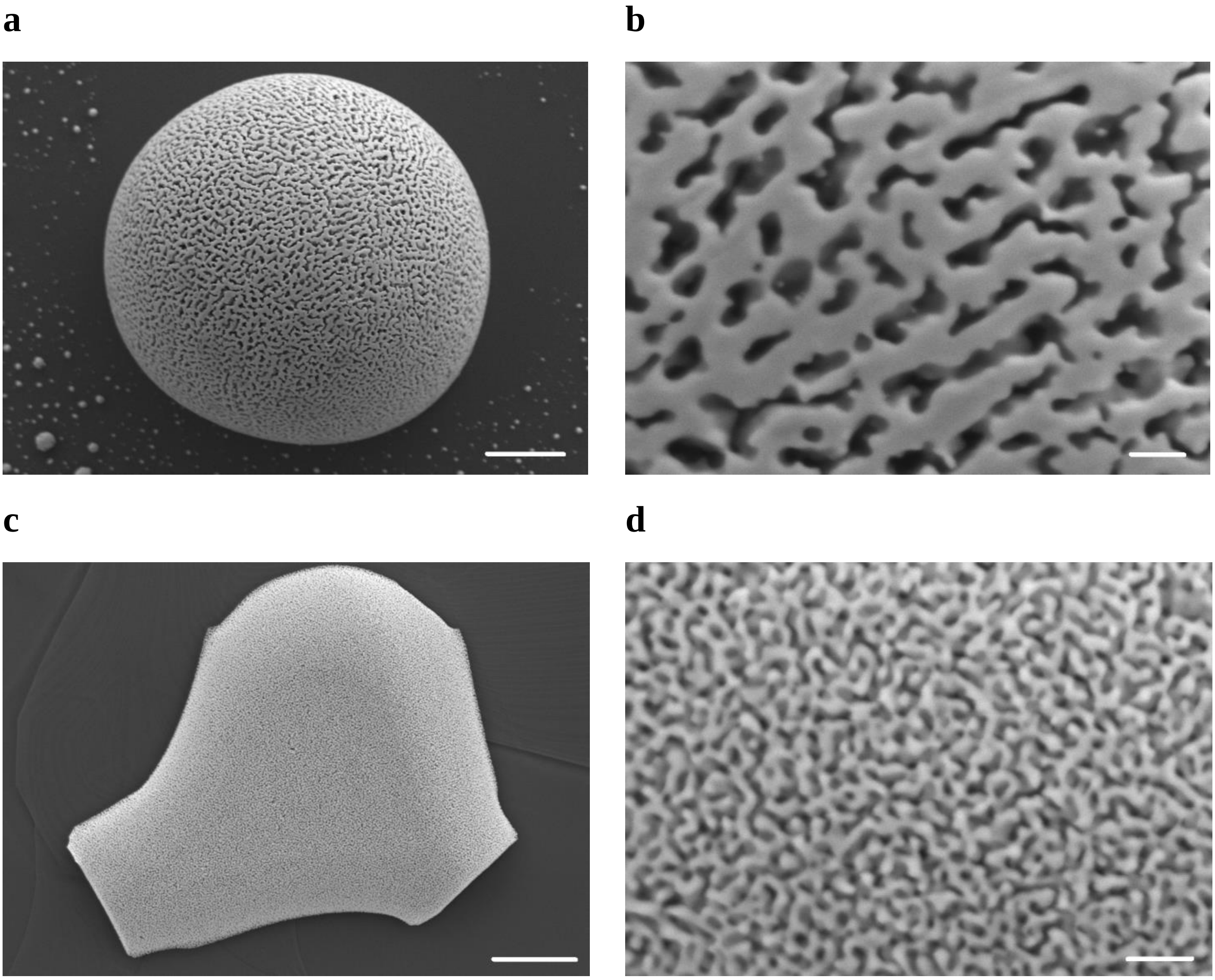

Supplementary Figure 2.HRSEM micrographs of the nanoporous structure in nanoporous gold prepared by eutectic decomposition or dealloying before thermal stability experiments. (**a**) Sample prepared by eutectic decomposition (scale bar, 1 μm). (**b**) High magnification of **a** (scale bar, 100 nm). (**c**) Sample prepared by dealloying (scale bar, 1 μm. (**d**) High magnification of **c** (scale bar, 100 nm).

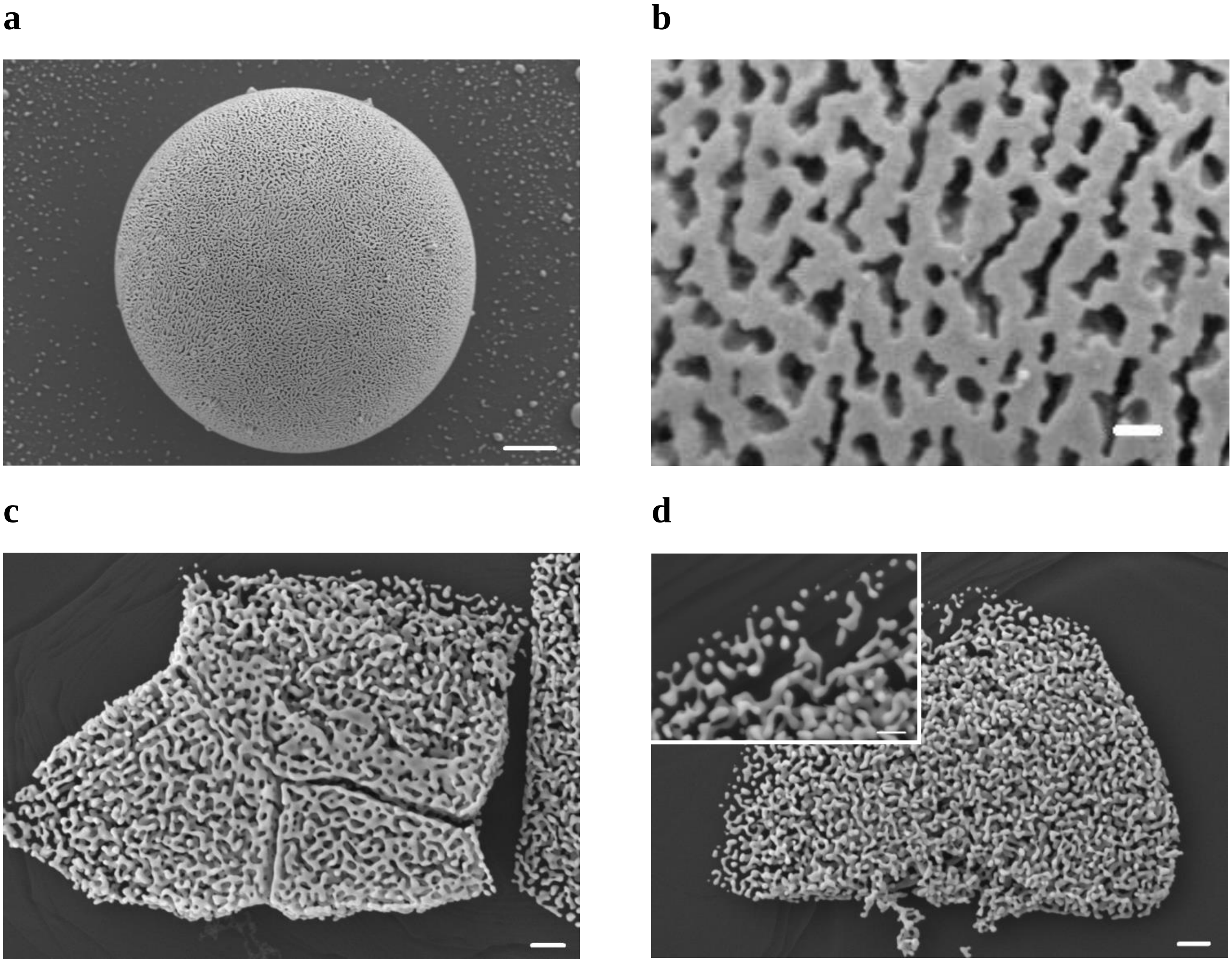

Supplementary Figure 3 HRSEM micrographs showing thermal stability of the nanoporous structure in nanoporous gold prepared by eutectic decomposition or dealloying. After preparation of the nanoporous gold, both samples were annealed at 200°C in vacuum for 15 min. (**a**) Sample prepared by eutectic decomposition retains the nanoporous structure with no damage (scale bar, 2μm). (**b**) High magnification of **a** (scale bar, 100 nm). (**c**) Sample prepared by dealloying shows cracks (probably from the grain boundaries) (scale bar, 200 nm). (**d**) Sample prepared by dealloying shows disintegration of material at the edges (scale bar, 200 nm, inset scale bar, 100 nm).

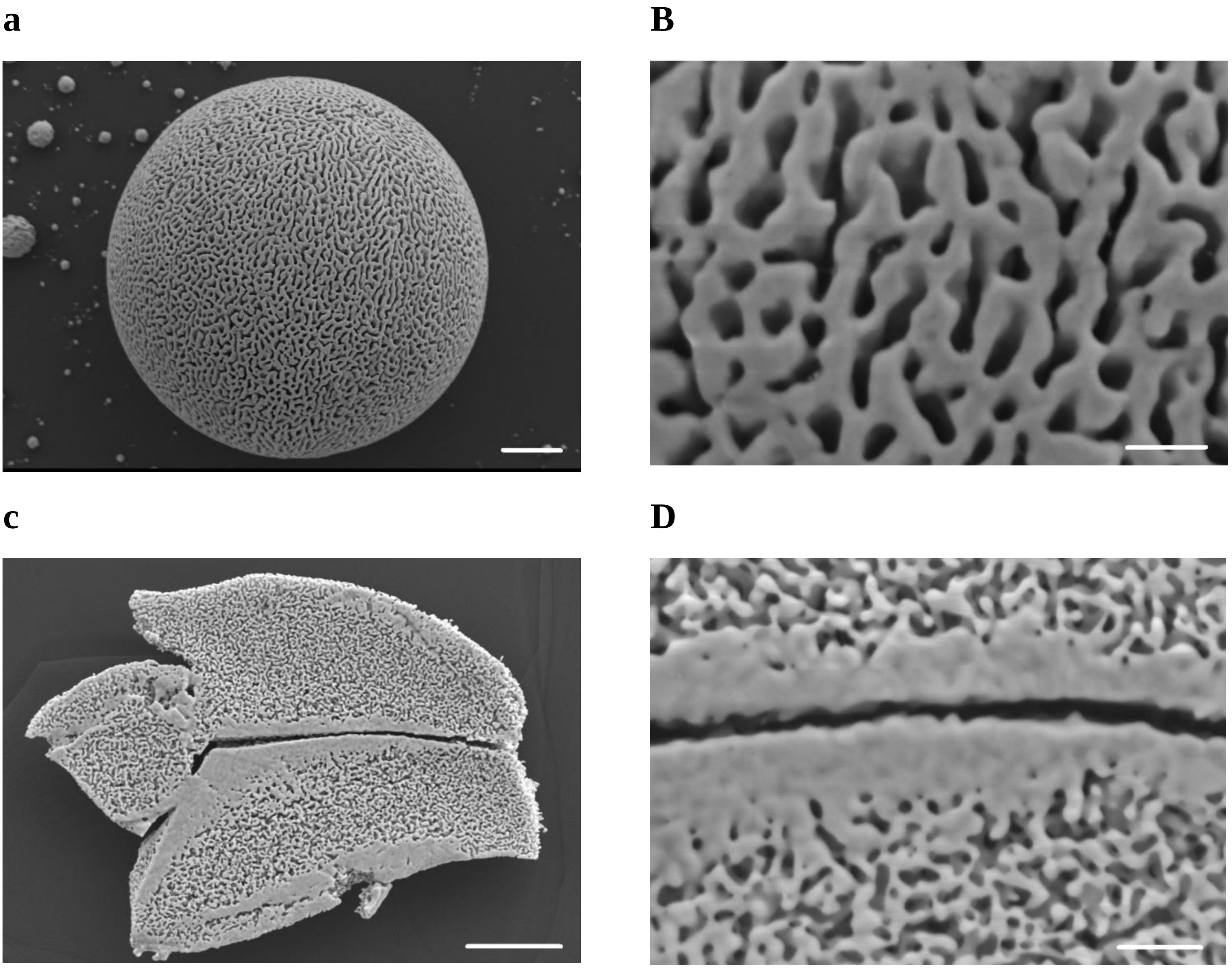

Supplementary Figure 4 HRSEM micrographs showing thermal stability of the nanoporous structure in nanoporous gold prepared by eutectic decomposition or dealloying. After preparation of the nanoporous gold, both samples were annealed at 200°C in vacuum for 45 min. (**a**) Sample prepared by eutectic decomposition retains the nanoporous structure with no damage (scale bar, 1 μm). (**b**) High magnification of **a** (scale bar, 200 nm). (**c**) Sample prepared by dealloying shows cracks (probably from the grain boundaries) and disintegration of material at the edges (scale bar, 1 μm). (**d**) High magnification of **c** (scale bar, 200 nm).

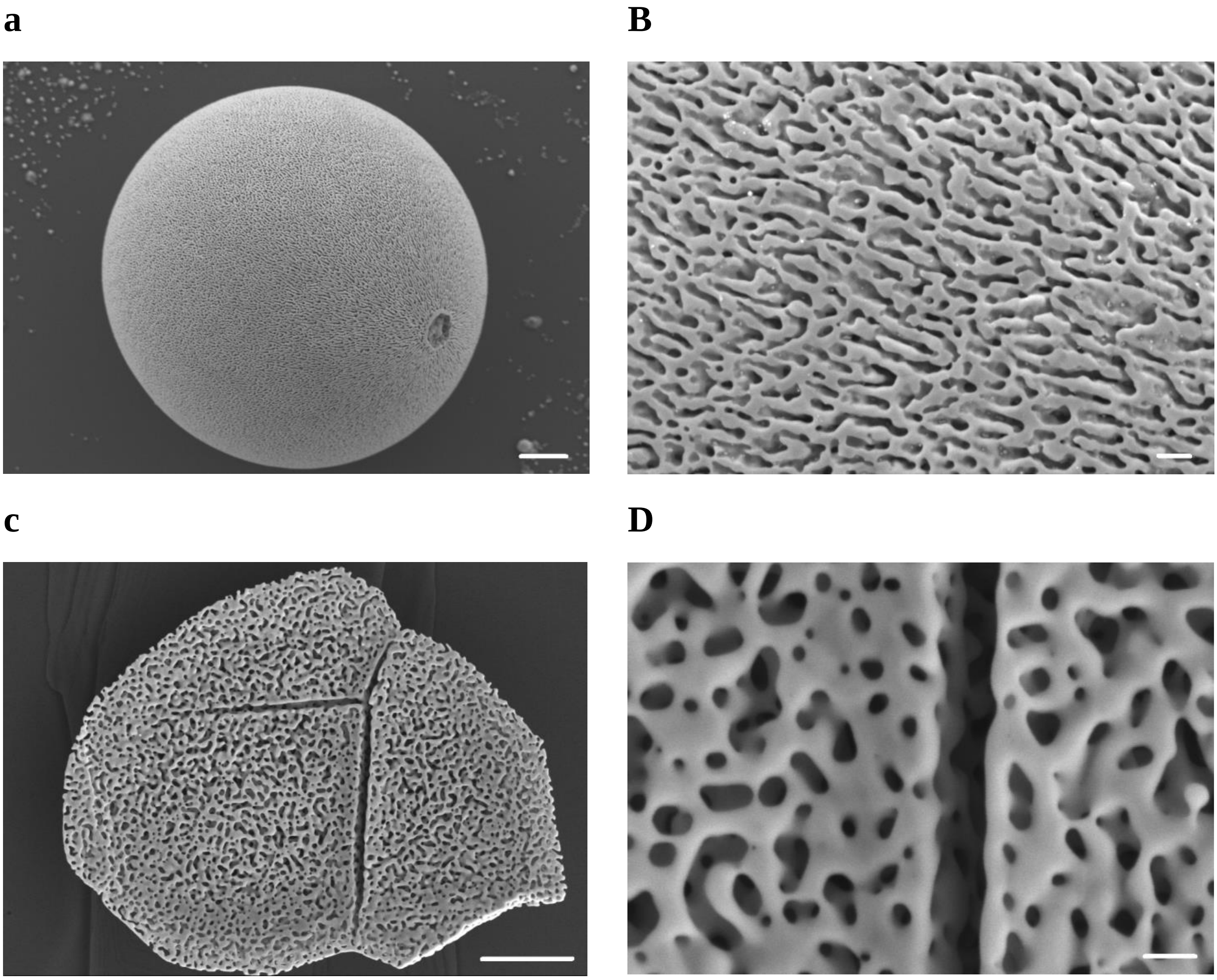

Supplementary Figure 5 HRSEM micrographs showing thermal stability of the nanoporous structure in nanoporous gold prepared by eutectic decomposition or dealloying. After preparation of the nanoporous gold, both samples were annealed at <u>250°C</u> in vacuum for <u>15 min</u>. (**a**) Sample prepared by eutectic decomposition retains the nanoporous structure with no damage (scale bar, 2 μm). (**b**) High magnification of **a** (scale bar, 200 nm). (**c**) Sample prepared by dealloying showscracks (probably from the grain boundaries) and coarsening (scale bar, 1 μm). (**d**) High magnification of **c** (scale bar, 100 nm).

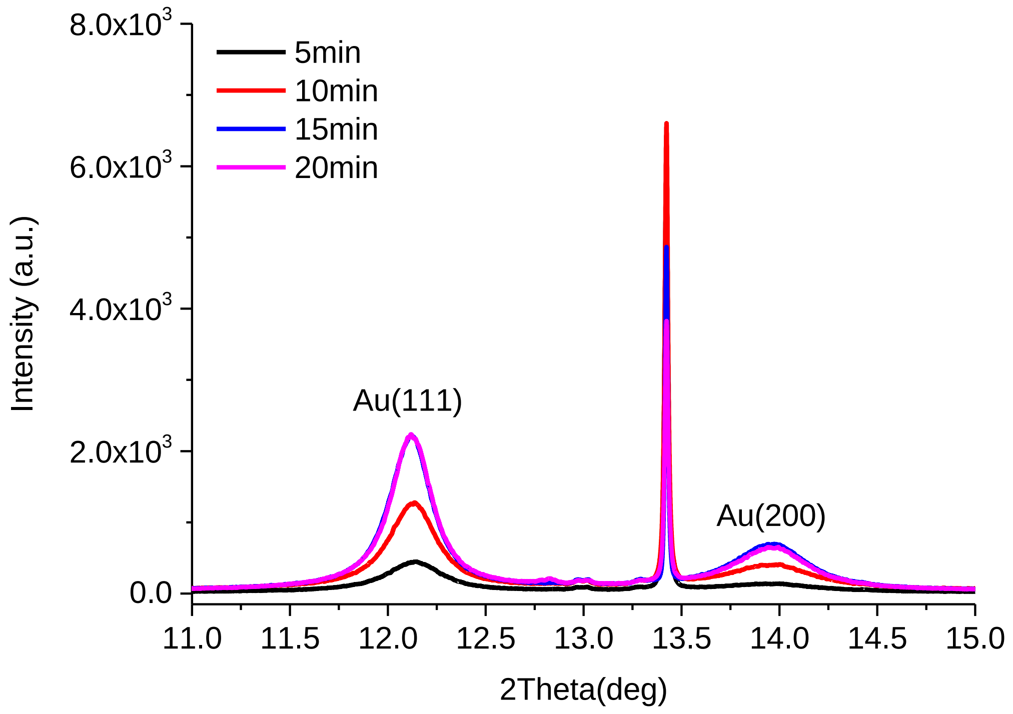

Supplementary Figure 6 High-resolution powder diffraction of *in-situ* dealloying of $AuAl_2$. Plots are at 5, 10, 15, and 20 min. For the Au(111) peak the coherence length after 20 min is 10 nm, whereas the original coherence length of the $AuAl_2$ intermetallic was 300 nm.

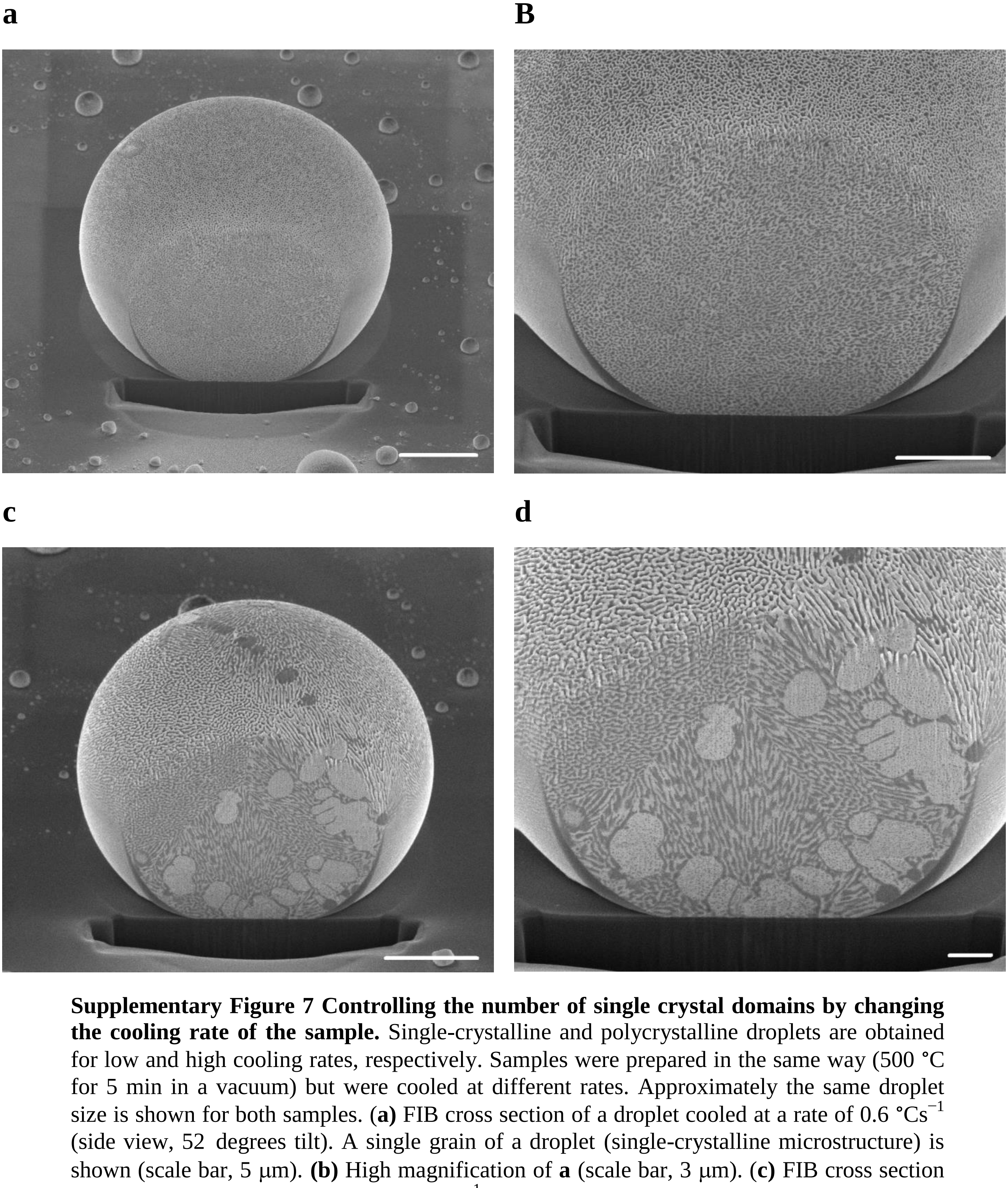

Supplementary Figure 7 Controlling the number of single crystal domains by changing the cooling rate of the sample. Single-crystalline and polycrystalline droplets are obtained for low and high cooling rates, respectively. Samples were prepared in the same way (500 °C for 5 min in a vacuum) but were cooled at different rates. Approximately the same droplet size is shown for both samples. (**a)** FIB cross section of a droplet cooled at a rate of 0.6 $°Cs^{-1}$ (side view, 52 degrees tilt). A single grain of a droplet (single-crystalline microstructure) is shown (scale bar, 5 μm). **(b)** High magnification of **a** (scale bar, 3 μm). (**c)** FIB cross section of a droplet cooled at a rate of 35 $°Cs^{-1}$ (side view, 52 degrees tilt). A polycrystalline microstructure is seen (and the ligament size is larger, as described in Fig. 4) (scale bar, 3 μm). **(d)** High magnification of **c** (scale bar, 1 μm).

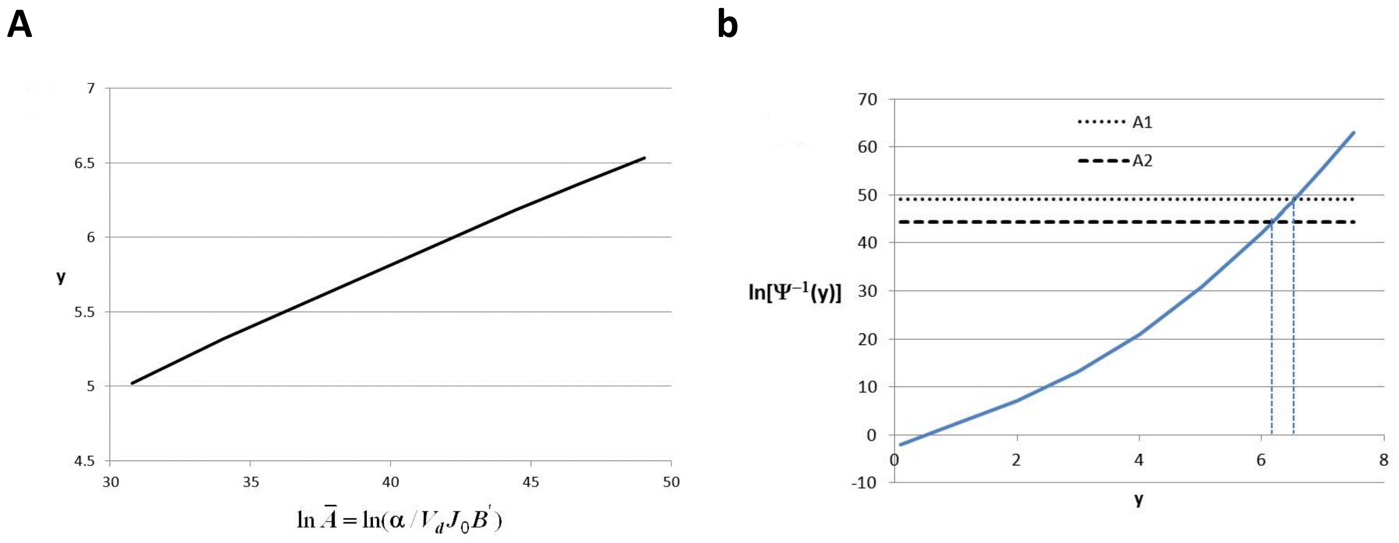

Supplementary Figure 8. Numerical solutions for Supplementary Note 2. (a) Solution of eq. 7, Supplementary Note 2: $\Psi(y) = \frac{\exp(-y^2)}{y} - \sqrt{\pi} erfc(y) = A$; (b) graphic illustration of the solution for $A_1 = 2 \cdot 10^{21}$ and $A_2 = 2 \cdot 10^{19}$; the solutions are y = 6.535 and y = 6.185, respectively.

Supplementary Table 1 Energy-dispersive X-ray spectroscopy (EDS) examination of the eutectic microstructure before and after Ge etching. The Ge concentration in the eutectic-like structure before etching was approximately 14 at.%, whereas after etching it was close to zero. 5−6 at% of Si was found in each spectrum, probably due to Si contamination from the wafer during focused ion beam (FIB) milling (the EDS examination was performed on a cross section of the droplet).

**at.%**	**C**	**O**	**Si**	**Ge**	**Au**
**Before Ge etching**	20.37	15.87	5.33	13.68	44.75
**After Ge etching**	21.37	14.88	5.95	0.37	57.43

Supplementary Table 2. Physical constants and calculated parameters for the Au-Ge system.

	**Units**	**Au/Ge**	**Ge**	**Au**
T_e	(K)	631		
T_f	(K)		1211.4	1337.33
$C_e(X = X_{Ge})$	(at.%)	28		
$D(T_\mathrm{e})$	($\mu m^2 s^{-1}$)	85		
v_{mol}	($10^{-5} m^3 mol^{-1}$)	1.1		
$\gamma_{\alpha\beta}$	(Jm^{-2})	0.174		
$\gamma_{s/l}^{i}$	(Jm^{-2})		0.145	0.1
f_i			0.31	0.69
ρ_i	(gcm^{-3})		5.3	19.3
ΔS_f^i	($JK^{-1}mol^{-1}$)		30.4975	9.3859
m_i	(K/at.%)		17	22
C_i^0	(at.%)		100	0.097
θ_i	(°)		54.8	33.4
a^L	($10^{-4}\mu m$)	2.5		
C_0	(at. fraction)	0.616		
P		0.025		
$\lambda^2 V$	($\mu m^3 s^{-1}$)	0.295		

Supplementary Table 3. Different sets of calculated parameters for the Au-Ge system.

	J_0 $10^{20}\mu m^{-3}s^{-1}$	ΔV_d μm^3	B' °C	α °Cs^{-1}	A 10^{20}	y_1	y_2	R_d μm	V μms^{-1}	Δt_{12} s	$\tau=R_d/V$ s
I	4	0.01	175	35	0.2	6.185	6.13	5	30	0.007	0.17
II	4	0.01	500	1	20	6.535	6.483	5	30	0.61	0.17
III	5	0.017	700	1	60	6.616	6.563	6.5	50	0.84	0.13
IV	6	0.04	700	0.5	336	6.741	6.691	10	30	1.55	0.3

Supplementary Note 1

Steady-state eutectic growth velocity

According to the JH theory [1-4], the steady-state growth of lamellar or rod eutectic structure is described by the following relationship between velocity *V*, structure spacing (ligament size) *λ*, and diffusion coefficient in the liquid phase *D*:

$$\lambda^2 V = \frac{Df\left(1-f\right)a^L}{C_0 P(f,p)}, \tag{1}$$

where $a^L = 2\left[\frac{\Gamma_\alpha \sin\theta_\alpha}{f m_\alpha} + \frac{\Gamma_\beta \sin\theta_\beta}{(1-f)m_\beta}\right]$, $C_0 = \frac{\rho_\alpha\left(C_e - C_\alpha^0\right) - \rho_\beta(C_\beta^0 - C_e)}{\rho_\alpha f + (1-f)\rho_\beta}$, C_i^0 is the concentration in the solid phase *i* at the eutectic temperature, $\Gamma_i = \gamma_{s/l}^i / \Delta S_f^i$ is the Gibbs-Thomson coefficient, $\gamma_{s/l}^i$ is the solid/liquid interface energy, ΔS_f^i is the entropy of fusion of the phase *i*, m_i is the slope of the liquidus line (defined as positive) in the α/β phase diagram corresponding to the *i* phase (*i* = α or β), and θ_α and θ_β are the contact angles at the three-phase junction [2], which can be derived from the surface tension balance:

$$\begin{cases} \gamma_{s/l}^\alpha \sin\theta_\alpha + \gamma_{s/l}^\beta \sin\theta_\beta = \gamma_{\alpha\beta} \\ \gamma_{s/l}^\alpha \cos\theta_\alpha = \gamma_{s/l}^\beta \cos\theta_\beta \end{cases}, \tag{2}$$

where $\gamma_{\alpha\beta}$ is the surface energy of the isotropic α/β interface. The function $P(f,p)$ is defined as [2]:

$$P(f,p)=\sum_{n=1}^{\infty}\frac{1}{(\pi n)^3}\left[\sin(\pi nf)\right]^2\frac{p_n}{1+\sqrt{1+p_n^2}}, \tag{3}$$

where $p_n=2\pi n/\bar{p}$, $\bar{p}=V\lambda/2D$ is the Peclet number. For low velocities ($\bar{p}<<1$ the case usually assumed in the JH treatment), the function (3) can be well approximated by the analytical expression [3]: $P\cong 0.3251\left[1-0.205exp\left(-24f_\alpha f_\beta\right)\right](f_\alpha f_\beta)^{1.63}$.

The physical constants taken from [5, 6] and calculated parameters for the Au-Ge system are presented in Supplementary Table 2. As can be determined from the last row of the table, for the eutectic structure spacing $\lambda=\left(70\div 100\right)$nm, the steady-state growth velocities are $V=\left(30\div 60\right)$ μms^{-1}.

Supplementary Note 2.

Heterogeneous nucleation rate in the Au-Ge eutectic system

The steady-state nucleation rate is deduced on the basis of classical kinetic theory [7, 8] by eq. (4) (main text):

$$J_0=\frac{bD}{d^2}\left(i^*\right)^{2/3}N_lZ, \tag{1}$$

where b=24 is a geometrical factor, $N_l = N_0/v_{mol}$ is the number density of molecules in the liquid, N_0 is the Avogadro number, v_{mol} is the molar volume of the liquid, $d=1/\left(N_l^{1/3}\sqrt{3}\right)$ is the intermolecular distance in liquid, i^* is the number of molecules in the critical fluctuation, and Z~0.01 is the Zeldovich factor. If we neglect the temperature dependence of the Au diffusion coefficient in the liquid, assume D = 8·10^{-11} m^{2}s^{-1} (as evaluated above), and use i^* =

$\Delta V^{*}N_{l}$ = 30÷50 where ΔV^{*} is the critical nucleus volume v_{mol} = 1.1·10^{-5}m^{3}mol^{-1}, then we can evaluate $J_0 = (4.4 \div 6.2)\cdot 10^{38} m^{-3}s^{-1}$.

The height of the heterogeneous nucleation barrier W^{*} can be deduced from the classical nucleation theory (CNT) [7, 8] or from the diffuse interface theory (DIT) [9] for nuclei of different shapes. In the CNT, sharp interfaces with constant interface energies are assumed. If the nucleus is considered as a solid droplet on the liquid/substrate surface with a contact angle of θ, the heterogeneous nucleation barrier is the following [7, 8]:

$$W_{het}^{CNT} = \kappa S(\theta)\frac{\left(\gamma_{Au/liq}\right)^{3}}{\left(\Delta g_{0}\right)^{2}} \quad , \qquad (2)$$

where Δg_0 is the volumetric free-energy change due to the transformation of liquid to solid, $\gamma_{\mathrm{Au/liq}}$ is the solid/liquid interface energy, $S(\theta) = (2 - 3cos\theta + cos^{3}\theta)/4$, and $\kappa = \frac{16\pi}{3}$. This barrier can be substantially lower than the barrier to homogeneous nucleation for θ < 90°.

The DIT is based on the assumptions that bulk properties exist at least at the center of critical fluctuations and that the distance between the surfaces of zero excess enthalpy and zero excess entropy is independent of cluster size [9]. The height of the homogeneous nucleation barrier for a droplet nucleus according to the DIT approach is written as

$$W_{\mathrm{hom}}^{DIT} = \kappa\delta^{3}\Delta g_{0}\Phi(\eta) \quad , \qquad (3)$$

where δ is the characteristic interface thickness, $\Phi = 2(1+q)\eta^{-3} - (3+2q)\eta^{-2} + \eta^{-1}$, $q = \sqrt{1-\eta}$, $\eta = \Delta g_0 / \Delta h_0$, and Δh_0 is the volumetric enthalpy difference between the solid and the liquid. For the heterogeneous nucleation the additional factor $S(\theta)$ should be added in (B3). The interface energy defined in the DIT as $\gamma_{eff} = -\delta\Delta g_0(\Phi/4)^{1/3}$ is temperature dependent through Δg_0 and η. Under equilibrium conditions, T = T_f: $W_{het}^{DIT} = \kappa S(\theta)\frac{\left(\gamma_{\infty}\right)^{3}}{\left(\Delta g_{0}\right)^{2}}$, where $\gamma_{\infty} = -\delta\Delta h_0$. Bearing in mind that the interface energies [5] have been calculated for

certain experimentally determined undercooling temperatures T_u, the parameter δ can be derived as $\delta = \gamma_{\exp}\Delta g_0(T_u)[\Phi(T_u)/4]^{1/3}$. For gold, T_u = 1107K [5], which yields δ_{Au}≈0.13 nm and γ_∞ = 0.145J m^{-2}; while for germanium T_u = 1004K, δ_{Ge}≈0.06 nm and γ_∞ = 0.199Jm^{-2}. At the eutectic temperature $\gamma_{Au}(631K) = 0.1$ Jm^{-2}, $\gamma_{Ge}(631K) = 0.145$ Jm^{-2} and the values used in the present calculations (Table 1).

The values of Δg_0 and $\eta = \Delta g_0 / \Delta h_0$ for crystallization of gold from the eutectic Au-Ge melt should be used in the expression (2) or (3). The free-energy change for the reaction L(X_{eut})→L'(X_L)+Au is:

$$\Delta G = \frac{X_L - X_{eut}}{X_L}\Delta g_f^{Au} - T\left[S_L^{conf}(X_{eut}) - S_L^{conf}(X_L)\right] + \Delta g^E(X_{eut}, X_L), \qquad (4)$$

where Δg_f^{Au} is the free energy of fusion of Au, $S_L^{conf}(X_{eut})$ and $S_L^{conf}(X_L)$ are the configuration entropies in the liquid at eutectic and liquidus concentrations, and $\Delta g^E(X_{eut}, X_L)$ is the excess free-energy change in the liquid due to the transformation. The first term in (4) is responsible for nucleation of a gold crystal, and the other two for supersaturation of the melt with Ge, which then provides nucleation of a Ge crystal. Using the first term as the driving force for the initial transformation of liquid to solid Au, we can write:

$$\Delta g_0 = \frac{X_L - X_{eut}}{X_L}\Delta g_f^{Au} \approx \frac{\Delta T}{m_{Au}X_{eut} + \Delta T}\Delta g_f^{Au} \equiv \varphi(\Delta T)\Delta g_f^{Au} \qquad (5)$$

The time t_1 required for heterogeneous nucleation of a first nucleus inside a droplet can be determined by integration of the nucleation rate (4, main text) with time:

$$\Delta V_d \int_0^{t_1} J_0 \exp\left(-\frac{W^*}{kT}\right)dt = 1 , \qquad (6)$$

where ΔV_d is the droplet volume appropriate for heterogeneous nucleation. For a droplet with a radius r, the volume appropriated for heterogeneous nucleation is ΔV_d~πr^{2}h (h is the near-substrate layer thickness); if, for example, r = 2 μm and h = 1 nm, then ΔV_d≈ 0.013 μm^3. Since the nucleation occurs over a narrow temperature interval [9], we can neglect the change of diffusion coefficient during the cooling and assume T≈const in the exponent's denominator, while the undercooling ΔT has to be considered as the time-dependent parameter $\Delta T = T_{eut} - T = \alpha t$, where α is the cooling rate. With these simplifications, integration results in the following expression:

$$\bar{A}^{-1} = \left[\frac{1}{y_1} e^{-y_1^2} - \sqrt{\pi} erfc(y_1) \right] \equiv \Psi(y_1), \quad (7)$$

where $y_1 = \frac{B^{'}}{\alpha t_1}$, $\bar{A} = \Delta V_d J_0 B^{'} / \alpha$, $B^{'} = \frac{\left[\kappa S(\theta) \gamma_{Au/liq}^3 \right]^{1/2} m_{Au} X_{eut}}{\Delta S_f^{Au} \left(T_f - T_{eut} \right) \sqrt{kT}} \cong Const$. An approximate expression for the function $\Psi(y_1) \approx e^{-y_1^2} / 2y_1^3$ can be used for $y_1 \geq 6$. A numerical solution of eq. (7) is illustrated in Supplementary Figure 8. As shown, a change of two orders of value in the product $\bar{A}$ results in variation in y_1 of only ~5.4%. Using the values of parameters for Au (Supplementary Table 2), we can evaluate the value of $B^{'} \approx 1580 \cdot S^{\frac{1}{2}}(\theta)$ °C and the undercooling value as $\Delta T = \alpha t_1 = B^{'} / y_1$. Maximum experimental values of undercooling for nucleation in metal drops (up to 50 μm diameter) are ~$0.18T_f$ [5]. Using T_{eut} = 631*K* as a melting temperature for the Au-Ge system we can evaluate $\Delta T_m = 114$ °C. The value $y_1 = (6.2 \div 6.5)$ (see below) corresponds to $B' = (707 \div 740)$ °C and $S(\theta) \approx 0.2$ $(\theta \approx 65°)$. For such values of $B^{'}$ the first nucleus appears after $t_1 \approx 3.5$ s for the cooling rate $\alpha = 35$ °Cs^{-1} and $t_1 \approx 3.5$ min for $\alpha = 35$ °Cmin^{-1}.

The average period between the first and the second nucleation events can be evaluated from the equation $\Psi(y_2) = 2\Psi(y_1)$. The exact numerical solution yields $y_2 = 6.130$ for $y_1 = 6.185$ ($\bar{A}$ = $2{\cdot}10^{19}$) and $y_2 = 6.483$ for $y_1 = 6.535$ ($\bar{A}$ = $2{\cdot}10^{21}$). The approximate solution $y_1 - y_2 \approx \ln 2 / (2 y_1)$ gives approximately the same values with an error of ~0.02%.

Then the period between two first nucleation events is as follows:

$$t_2 - t_1 \equiv \Delta t_{12} = \frac{B^{'}}{\alpha} \frac{(y_1 - y_2)}{y_1 y_2} \approx \frac{B^{'}}{\alpha} \frac{ln2}{2y_1^3} = (1.2 \div 1.5) \cdot 10^{-3} \left(\frac{B^{'}}{\alpha} \right) \quad (8)$$

For determination of values of $B^{'}$, the period Δt_{12} is evaluated as $\Delta t_{12} = (2.4 \div 3.2) 10^{-2}$ s for $\alpha = 35$ °Cs^{-1} and $\Delta t_{12} = (1.4 \div 1.9)$ s for $\alpha = 35$ °Cmin^{-1}. Several reasonable sets of calculated parameters are presented in Supplemetary Table 3.

Supplementary References

1. K. A. Jackson, J.D.H., *The Dendrite-Eutectic Transition.* Trans. AIME 1966. 236: p. 1129-1142.
2. Trivedi, R., P. Magnin, and W. Kurz, *Theory of eutectic growth under rapid solidification conditions.* Acta Metallurgica, 1987. 35(4): p. 971-980.
3. Kurz, W. and R. Trivedi, *Eutectic growth under rapid solidification conditions.* Metallurgical Transactions A, 1991. 22(12): p. 3051-3057.
4. Karma, A., *Phase-field model of eutectic growth.* Physical Review E, 1994. 49(3): p. 2245.
5. Turnbull, D., *Correlation of Liquid-Solid Interfacial Energies Calculated from Supercooling of Small Droplets.* The Journal of Chemical Physics, 1950. 18(5): p. 769-769.
6. Dinsdale, A.T., *SGTE data for pure elements.* Calphad, 1991. 15(4): p. 317-425.
7. Kelton, K., *Crystal nucleation in liquids and glasses.* Solid state physics, 1991. 45: p. 75-177.
8. Debenedetti, P.G., *Metastable liquids: concepts and principles*1996: Princeton University Press.
9. Gránásy, L., *Diffuse interface theory of nucleation.* Journal of non-crystalline solids, 1993. 162(3): p. 301-303.
